# Real-time plant health assessment via implementing cloud-based scalable transfer learning on AWS DeepLens

**DOI:** 10.1371/journal.pone.0243243

**Published:** 2020-12-17

**Authors:** Asim Khan, Umair Nawaz, Anwaar Ulhaq, Randall W. Robinson

**Affiliations:** 1 The Institute for Sustainable Industries and Liveable Cities (ISILC), College of Engineering and Science, Victoria University, Melbourne, Australia; 2 Department of Electrical Engineering, Namal Institute Mianwali, Mianwali, Pakistan; 3 School of Computing and Mathematics, Charles Sturt University, Port Macquarie, NSW, Australia; Taipei Medical University, TAIWAN

## Abstract

The control of plant leaf diseases is crucial as it affects the quality and production of plant species with an effect on the economy of any country. Automated identification and classification of plant leaf diseases is, therefore, essential for the reduction of economic losses and the conservation of specific species. Various Machine Learning (ML) models have previously been proposed to detect and identify plant leaf disease; however, they lack usability due to hardware sophistication, limited scalability and realistic use inefficiency. By implementing automatic detection and classification of leaf diseases in fruit trees (apple, grape, peach and strawberry) and vegetable plants (potato and tomato) through scalable transfer learning on Amazon Web Services (AWS) SageMaker and importing it into AWS DeepLens for real-time functional usability, our proposed DeepLens Classification and Detection Model (DCDM) addresses such limitations. Scalability and ubiquitous access to our approach is provided by cloud integration. Our experiments on an extensive image data set of healthy and unhealthy fruit trees and vegetable plant leaves showed 98.78% accuracy with a real-time diagnosis of diseases of plant leaves. To train DCDM deep learning model, we used forty thousand images and then evaluated it on ten thousand images. It takes an average of 0.349s to test an image for disease diagnosis and classification using AWS DeepLens, providing the consumer with disease information in less than a second.

## 1 Introduction

The effects of plant disease on quantitative and qualitative production [1] are devastating, resulting in a striking blow to farmers, traders and consumers. A 14.1% relative disease loss across all crops was observed in a US-based study conducted by the U.G.A. Center for Agribusiness and Economic Growth [2]. A description of losses due to plant disease reported by the University of Georgia Extension in the 2017 Georgia Farm Gate Value Study (AR-18-01) [2].

Traditionally farmers detect and diagnose plant diseases through their observations and rely upon the opinions of local experts and their past experiences. An expert can determine whether or not a plant is healthy [3]. If a plant is found unhealthy, noticeable symptoms on its leaves and fruits are observed and reported. Diagnosis of plant disease incorporates a substantially high degree of difficulty through visual examination of the symptoms on plant leaves. Because of this challenge and the huge number of grown plants and their existing phytopathological issues, even qualified agronomists and plant pathologists sometimes struggle to accurately identify particular diseases and are consequently driven to wrong assumptions and remedies [4]. Practical plant health assessment and diseases diagnosis can improve product quality and prevent production loss. Early detection and classification of crop disease are significant to secure the specific species production [5]. Various research studies have found that early detection of plant diseases is crucial as over the period, diseases start affecting the growth of their species, and their symptoms appear on the leaves [6]. When a plant got infected by a specific disease, and then significant symptoms are shown on the leaves, which help in the identification and classification of that particular disease [7]. It is therefore essential to control and assess disease outspread [8]. A specific fungus or bacterium is frequently associated with the colour, scale, form, and margins of spots and blight (lesions). Many fungi develop disease “signs”, such as mould growth or fruiting bodies that appear in the dead area as dark specks. Early stages of bacterial infections that develop during humid weather on leaves or fruits sometimes appear as dark and water-soaked spots with a separate margin and often a halo, a lighter-coloured ring around the site. As in peach plant, for instance, the decayed area is small and looks similar in appearance to neighbouring healthy tissue at an early stage; therefore, it is tough to detect diseases [9].

In the exploration of the agricultural field, technology plays a vital role. With the use of various machine learning and image processing techniques, researchers are trying to explore plant disease detection and classification. It is difficult, time-consuming and unreliable to detect plant diseases manually. Since a health evaluation is tedious and time-consuming for an individual plant in a large plot, this testing procedure is replicated over time [3]. A single plant may have different diseases having the same pattern of symptoms; moreover, various conditions of the plant show similar signs and symptoms [10], making it challenging to identify the specific disease. For instance, the key Grapevine Yellow (GY) symptoms are very common and outstanding in late summers, such as leaf discolouration, bunch drying and abnormal wood ripening, allowing GY to be recognized and, by and large, differentiated from other grapevine disorders that may exhibit similar alterations (e.g. leafroll or direct damage due to feeding of leafhopper). However, the expression of symptoms among different GYs is very standardized, so symptomatology is not helpful to distinguish one GY from another. Since phytoplasmas are poorly transmitted by grafting on woody plants and because the symptomatic response induced by various GY agents in Baco 22A is the same, even indexing on the hybrid Baco 22A, used in the past, did not help much [11, 12].

Machine learning (ML) [13] algorithms are serving a lot in the process of classification and identification of plant diseases automation. ML helps in monitoring of health assessment of plant and predicting diseases in the plant at early stages [7]. With the time progression, new ML models evolved, such as SVM [14], VGG architectures [15], R-FCN [16], Faster R-CNN [17], SDD [18] and many others. The researchers used them for their experiments in the field of recognising and classifying images. Some of those are used in automation of Agriculture systems [6].

The advancement in deep learning (DL) [19] has provided promising results and solutions in crop disease diagnosis and classification. Islam et al., [20] presented the integration of machine learning and image processing for the detection and classification of leaf disease images. They developed an SVM model for potato disease detection and used potato leaves dataset, consisting of healthy leaves and diseased leaves. For performance, they used performance parameters such as accuracy, sensitivity, recall and F1-score. Dubey et al., [21] came up with an image processing technique by using the K-Means algorithm for the detection and classification of apple fruit disease and then used multiclass SVM for training and testing images. Al-Amin et al., [7] trained their model for potato disease detection through Deep CNN, and they computed performance for analysing the result using parameters such as recall, precision and F1-score. This model achieved an accuracy of 98.33% in experiments. According to Sladojevic et al., [22] to learn features, CNN must be trained on a large dataset of a large number of images. They developed a CNN model for classification of leaves diseases of apple and tomato plants and the experimental accuracy findings of their research for numerous diseases trial with an accuracy of 96.3%. Miaomiao et al., [23] presented an effective solution for grape diseases detection as they mentioned that two entirely different basic models integrated, it would be more useful to obtain remarkable results and improve the accuracy of detection. Therefore, they proposed a UnitedModel based on the integration of GoogLeNet with ResNet, whereas GoogLeNet raises the total units for all layers of a network and ResNet to increase the total number of layers in a network. Ye Sun et al., [9] developed a model based on structured-illumination reflectance imaging (S.I.R.I.) for identification of peach fungal diseases. In their work, CNN and three image classification methods used for processing of ratio images, alternating component (AC) images and direct component (DC) images to detect the diseases and area of peach. As a result, they found that A.C. images performance is better than D.C. images in peach diseases detection and ratio images gave a high accuracy rate. Hyeon Park et al., [24] developed a CNN network of two convolutional and three fully connected layers, for disease detection in the strawberry plant. They worked on a small dataset of leaves images consisting of healthy leaves and a powdery mildew strawberries disease class. Xiaoyue et al., [25] worked on four typical grapes diseases, and for detection, they proposed a Faster DR-IACNN detector, based on deep learning. They reported that their proposed detector automatically detects the diseased spots on grapes leaves, thus giving an excellent result for the detection of diseases in real-time. In order to detect leaves diseases in vegetables, Zhang et al., [26] come up with an RGB model colours based three channels CNN. Konstantinos et al., [4] detected and classified 25 plant diseases by using different CNN based architectures. They trained and tested their model on the open-source dataset named PlantVillage. However, the results obtained in terms of accuracy may differ from using the same dataset for both training and testing purposes.

According to the above-discussed studies, CNN [27, 28] always played a significant role and is widely used in the detection and classification of different plant diseases and provided agreeably results. There were some limitations, however, such as a lack of usability due to hardware complexity problems, minimal scalability, inefficiency and minimal real-time inferences in real-world operational use.

The recent development in cloud-based services and efficient deep learning has motivated us to devise a practical and scalable solution to agricultural problems, and this paper lies in the similar domain. We found that most of the images in the PlantVillage dataset are either white or grey background; however, the real-world situation is different and may contain other colours in the background. Thus model trained only on uniform background colour may result in low accuracy or wrong prediction. Therefore, to address this research issue, we used a combination of publically available PlantVillage dataset [29] and images collected from Tarnab Farm (an agriculture research institute, Pakistan) real-cultivation environment to achieve high accuracy and a robust model. For training and testing, we used AWS SageMaker, a Cloud-based environment for our proposed model known as DeepLens Classification and Detection Model (DCDM) to identify and classify various fruits and vegetables leaves diseases, based on Deep Convolutional Neural Network (DCNN) [30]. After completion of training DCDM, it was deployed in the Internet of Things (IoT) device known as AWS DeepLens to make it a scalable and efficient real-time classification and identification model. AWS Deeplens is DL based high definition (H.D) video camera with 4 Mega-Pixel sensors for ML related projection integration and implementation.

**Fig 1:**
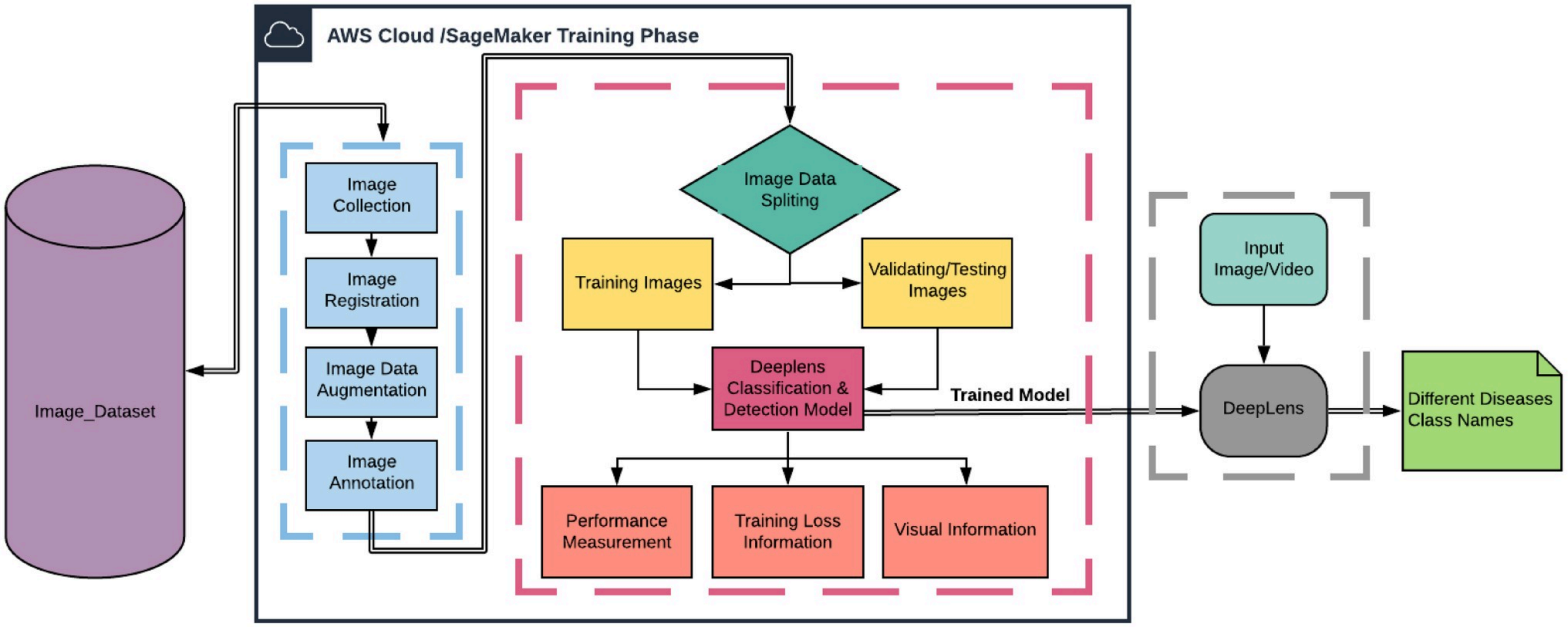
The data flow diagram of the DCDM that illustrates the process of our proposed disease diagnosis.

With our DCDM, we evaluated seven different CNN architectures using accuracy results and computation time. Those CNN architectures include ResNet-50 [31], AlexNet [32], VGG-16 [15], VGG-19 [15], DenseNet [33], SqueezeNet [34] and DarkNet [35]. All these architectures were trained and tested keeping the environment constantly. Our DCDM model out-performed all other architectures in terms of computation time as well as performance-wise. It obtained an average accuracy rate of 98.78% on test images. Our findings are the first step towards a system based on an AWS DeepLens camera for plant disease diagnosis. Moreover, in our work, we also extracted feature maps [22] of an input image after passing through the CNN model and applied filters to visualise the activations through the CNN layers [36]. The overall flow of the proposed DCDM model is illustrated in Fig 1.

The rest of the paper is organised as follow: Section 2 explains the materials and methodology, including pre and post-processing datasets, describing the proposed CNN model, AWS DeepLens transfer learning, and performance assessment. A detailed overview of experimental results is given in section 3. Section 4 introduces the discussion, while Section 5 offers recommendations for conclusion and future work, followed by references part.

## 2 Materials and methodology

**Fig 2:**
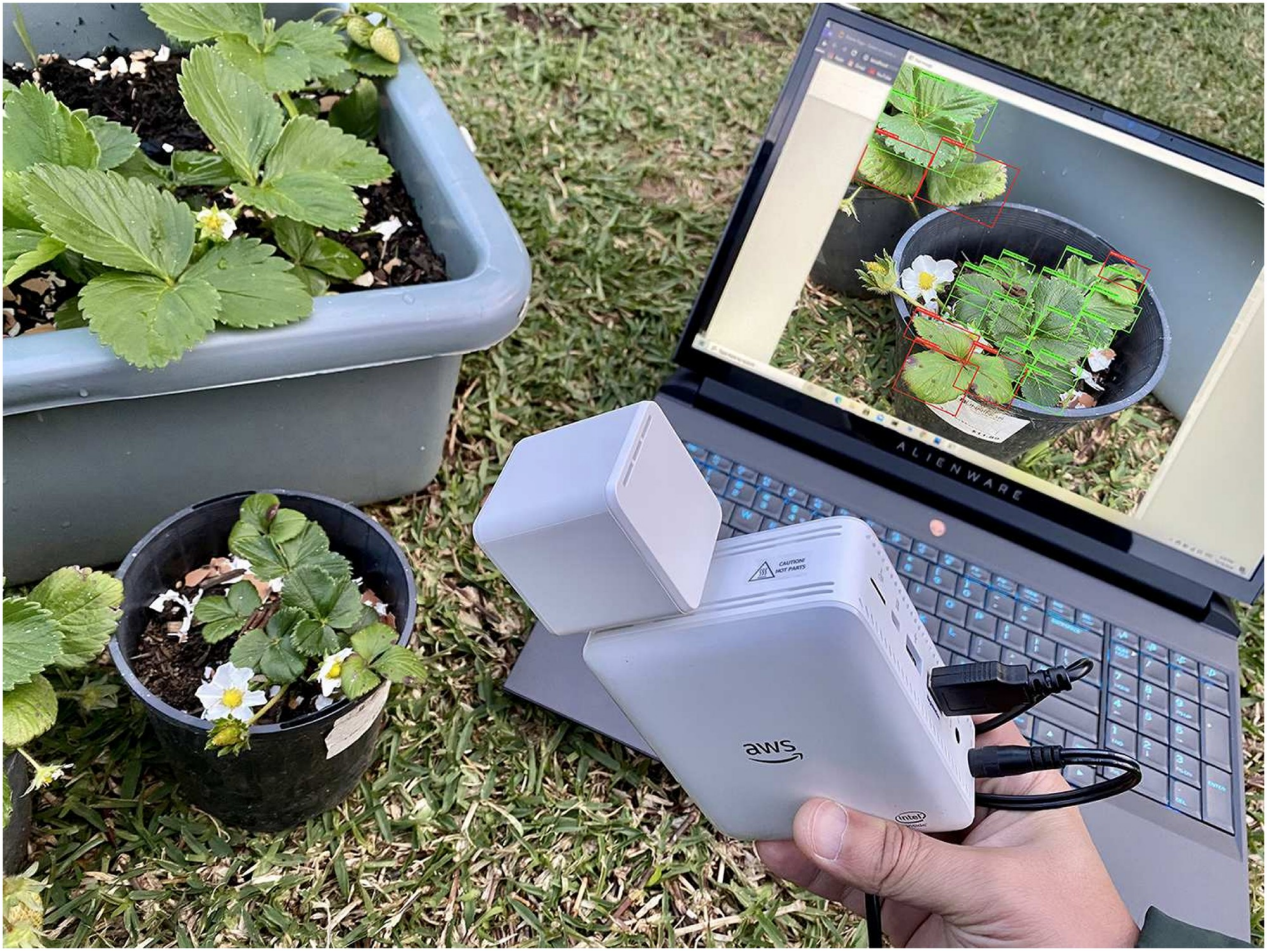
Identification & classification of strawberry plant leaf disease by AWS DeepLens in real-time.

The development process of the DCDM model for plant leaves disease detection, and classification involved various stages, i.e. starting with data collection along with data pre-processing and preparation, training model in AWS Cloud (SageMaker Studio) [37] and implementing in AWS DeepLens for inferences purpose. A strawberry plant is chosen for real-time disease assessment shown in Fig 2.

### 2.1 Dataset preparation

**Fig 3:**
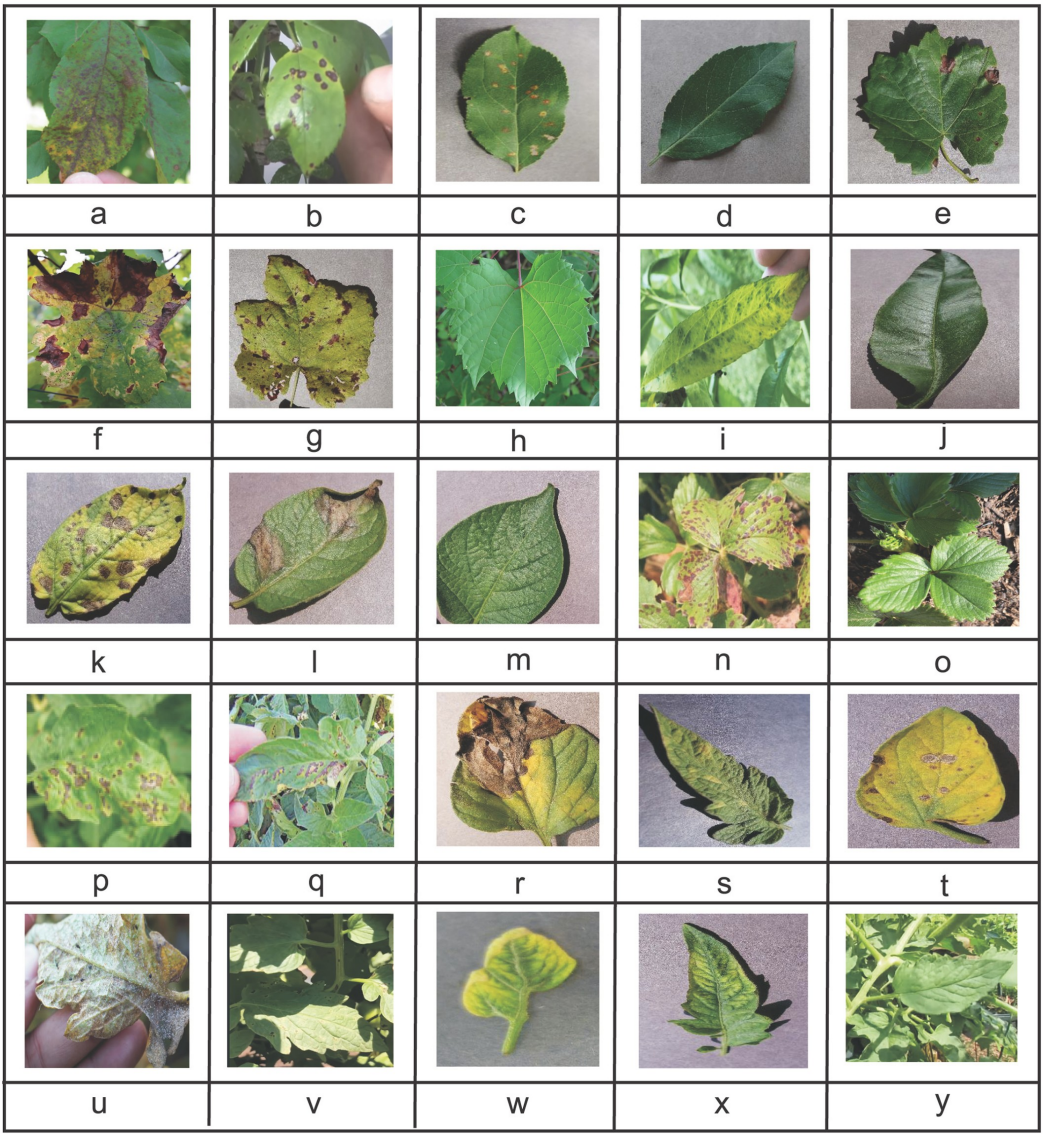
Sample images from dataset: (a). Apple Scab, (b). Black Rot, (c). Cedar Apple Rust, (d). Apple Healthy, (e). Grape Black Rot, (f). Grape Esca, (g). Grape Leaf Blight, (h). Grape Healthy, (i). Peach Bacterial Spot, (j). Peach Healthy, (k). Potato Early Blight, (l). Potato Late Blight, (m). Potato Healthy, (n). Strawberry Leaf Scorch, (o). Strawberry Healthy, (p). Tomato Bacterial Spot, (q). Tomato Early Blight, (r). Tomato Late Blight, (s). Tomato Leaf Mold, (t). Tomato Septoria Leaf Spot, (u). Tomato Spider Mites, (v). Tomato Target Spot, (w). Tomato Leaf Curl Virus, (x). Tomato Mosaic Virus, (y). Tomato Healthy. From PlantVillage: (c), (d), (e), (g), (j), (k), (l), (m), (r), (s), (t), (w) and (z). From Tarnab Farm: (a), (b), (f), (h), (i), (n), (o), (p), (q), (u), (v) and (y).

We used around 50,000 of plant leaves images (including both healthy and infected leaf images for fruit trees and vegetable plants) from local farmlands and publicly available dataset known as PlantVillage [29]. The dataset was categorised into different classes and assigned labels where each label is representing either a plant-leaf disease class or a healthy plant (leaf). A sample image for each class label shown in Fig 3.

### 2.2 Data augmentation

**Fig 4:**
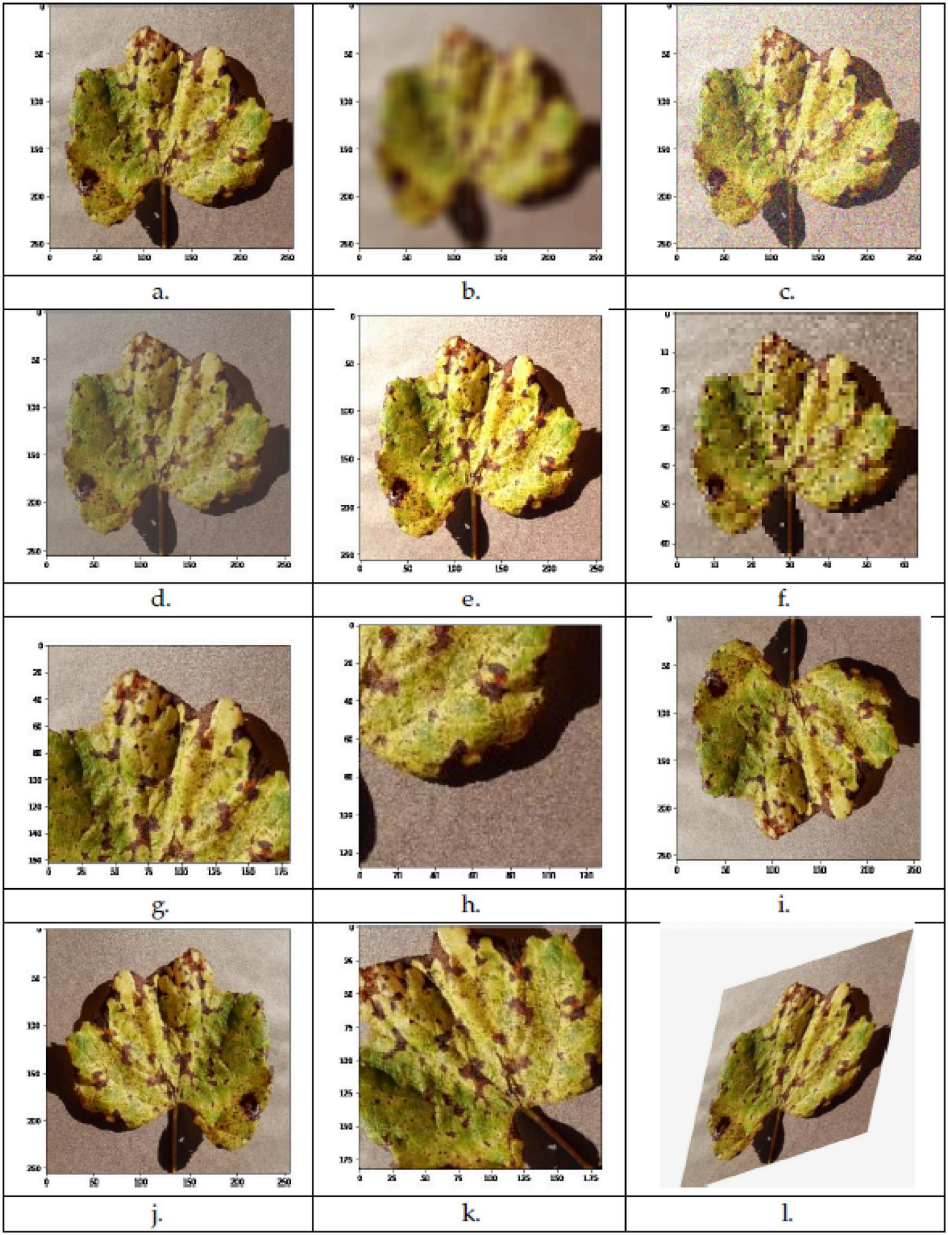
Data augmentation technique examples: (a). Original Image, (b). Blur, (c) Random Gaussian Noise, (d). Random Contrast, (e). Random Bright, (f). Scale Proportionality, (g). Random Crop, (h). Deterministic Crop, (i). Vertical Flip, (j). Horizontal Flip, (k). Rotate Without Padding, (l). Y-Sheared.

A large number of images are used to train a DCNN model to achieve highly precise prediction and accuracy. In our case, some of the plants leaves disease classes had fewer images in number; therefore, the process of data augmentation (technique) applied to those limited number of image diseases classes. The process of data augmentation [38] provided us with new images from our existing images. Different augmentation techniques like blurriness, rotation, flipping (horizontal and vertical), shearing (horizontal and vertical), and the addition of noise were applied accordingly. An illustration of different augmentation techniques shown in Fig 4. By using this technique, the number of images in our dataset increased, which is essential for obtaining more accurate results after the training stage of CNN.

### 2.3 Image registration and classes annotation

After completion of data augmentation process, we had to re-register the images in the same dimensions, as we used two types of a dataset having different dimensions. Image registration is an essential step in image processing whenever two or more images are processed and analysed [39]. It is a method of overlaying images (two or more) of the same scene taken at different times, from different points of view and/or from different sensors. It aligns two images (reference and sensed images) geometrically [40, 41]. We resized all the images into 272 x 363 pixels and annotated all the images before putting image as an input to any model/network for pre-training CNN structures. The classes of leaf diseases for fruits and vegetables that we used in our training and testing dataset are listed in the Table 1 with both regular and botanical names.

**Table 1:** The dataset for leaf disease classes.

Class No.	Pant Name	Plant Botanical Name	Disease Name	Disease Botanical Name	Total Images
1	Apple	Malus domestica	Scab	Venturia inaequalis	1830
2	Apple	Malus domestica	Black rot	Botryosphaeria obtusa	1821
3	Apple	Malus domestica	Cedar apple rust	Gymnosporangium juniperivirginianae	1675
4	Apple (Healthy)	Malus domestica			1725
5	Grapes	Vitis vinifera	Black rot	Guignardia bidwellii	2180
6	Grapes	Vitis vinifera	Esca	Phaeomoniella chlamydospora	1383
7	Grapes	Vitis vinifera	Leaf blight	Pseudocercospora vitis	2075
8	Grapes (Healthy)	Vitis vinifera			1823
9	Peach	Prunus persica	Bacterial spot	Xanthomonas campestris	2297
10	Peach (Healthy)	Prunus persica			1860
11	Potato	Solanum tuberosum	Early blight	Alternaria solani	2000
12	Potato	Solanum tuberosum	Late blight	Phytophthora infestans	2000
13	Potato (Healthy)	Solanum tuberosum			1652
14	Strawberry	Fragaria spp.	Leaf scorch	Diplocarpon earlianum	2237
15	Strawberry (Healthy)	Fragaria spp.			1856
16	Tomato	Lycopersicum esculentum	Bacterial spot	Xanthomonas campestris pv. Vesicatoria	2135
17	Tomato	Lycopersicum esculentum	Early blight	Alternaria solani	2257
18	Tomato	Lycopersicum esculentum	Late blight	Phytophthora infestans	1909
19	Tomato	Lycopersicum esculentum	Leaf mold	Fulvia fulva	2252
20	Tomato	Lycopersicum esculentum	Septoria leaf spot	Septoria lycopersici	1871
21	Tomato	Lycopersicum esculentum	Spider mites	Tetranychus urticae	1675
22	Tomato	Lycopersicum esculentum	Target spot	Corynespora cassiicola	1604
23	Tomato	Lycopersicum esculentum	Leaf curl virus		3852
24	Tomato	Lycopersicum esculentum	Mosaic virus	Tomato mosaic virus	2374
25	Tomato (Healthy)	Lycopersicum esculentum			1653

### 2.4 CNN and DeepLens Classification and Detection Model (DCDM)

A typical CNN consists of various layers. Each layer consists of multiple nodes with some activation function attached. The first layer is the input layer that takes input data, whereas, the last layer is the output layer that generates output. A random number of layers exists between the input and output layer, referred to as hidden layers (i.e. convolutional or convo, pooling, dense or fully connected and softmax layer) [27, 28]. If CNN contains two or more than two hidden layers, it is known as Deep Convolutional Neural Network (DCNN) [30].

We designed our DCDM using deep learning TensorFlow framework [42] and Keras [43] library. Keras is an open-source deep-learning library used to perform different deep learning applications. We used it for the implementation of DCDM architecture, inspired by Visual Geometry Group (VGG) Neural Networks. It is an advanced model of object-recognition supporting up to 16-19 weight layers [15]. Constructed as a deep CNN, VGG also out-performs baselines outside of ImageNet on several tasks and datasets. There are two variants of VGG Neural Networks namely VGG-16, which comprises of 16 convolutional layers and VGG19 comprises of 19 convolutional layers. VGG is also one of the most used architectures for image recognition today. This architecture uses filters of the same width and height for all the convolutional layers. The architecture of VGG-16 and VGG-19 out-performed than the other state-of-the-art architectures like ResNet-50, DenseNet, InceptionVNet [44] as they converge very quickly and score over 90% accuracy during the first epochs of training. The VGG-19 architecture consists of roughly about 138 million parameters [45] while VGG-16 has less number of parameters due to less number of layers, however, a large number of parameter makes computationally expensive for training purpose.

Our prposed architecture has the same sequential structure as of VGG Neural Network but with some less number of layers, thus, the numbers of parameters are extensively low, which makes it computationally less expensive and fast.

**Fig 5:**
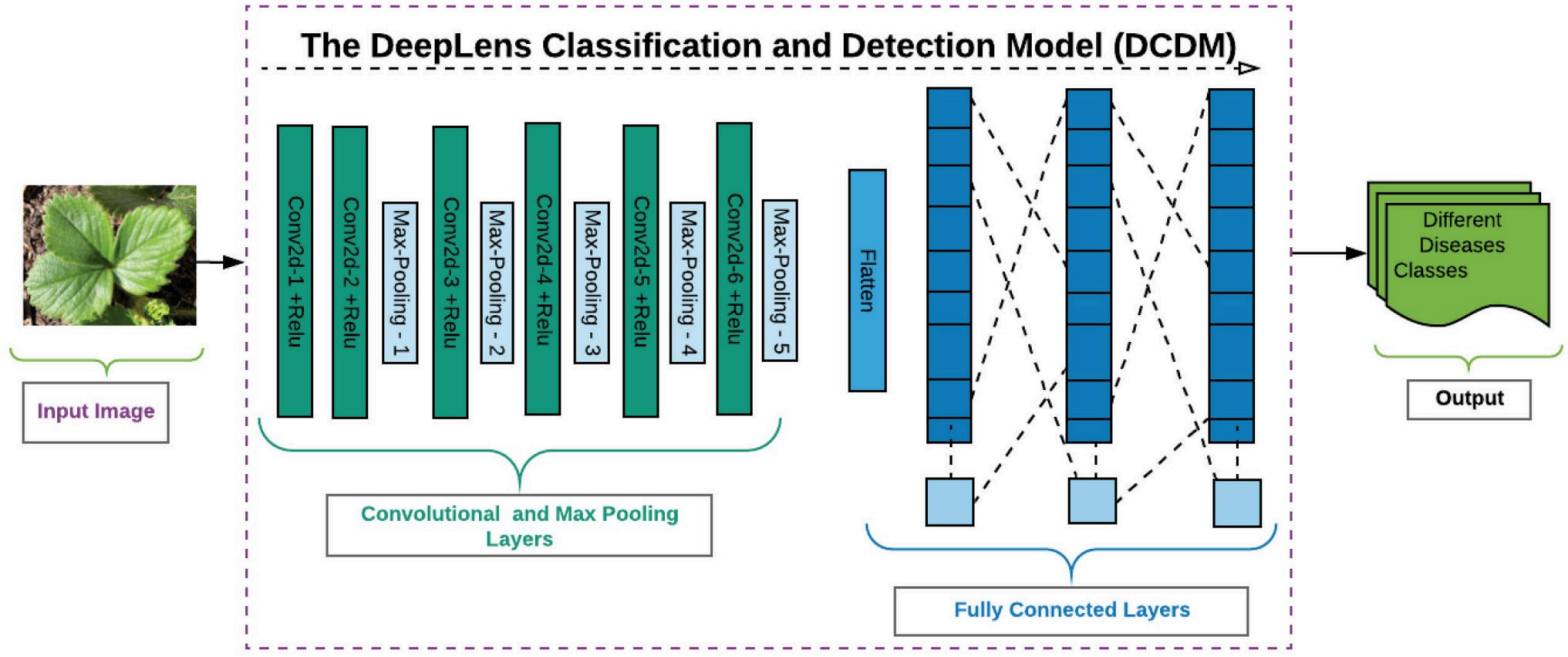
The representation of DeepLens Classification and Detection Model (DCDM) architecture.

Our DCDM architecture contains a total of nine layers with six convolutional layers and three fully connected layers shown in Fig 5. Convolutional layers are having non-linearity activation units following by max-pooling layers. The non-linearity activation is often used with convolutional layers. This activation is also known as a ramp function which has a shape of the ramp and transfers the output once it is a positive value; else it results in 0. The last layer, which is also known as a SoftMax layer comprising of 25 nodes in the output layer where each node specifies an individual class of our dataset.

The details of these layers are described below and shown in Table 2.

**Table 2:** The summary Of DCDM layered architecture.

Layer (type)	Output Shape	Param #
conv2d (Conv2D)	(None, 272, 363, 64)	1792
*conv*2*d*_1(*Conv*2*D*)	(None, 272, 363, 64)	36928
*max*_*pooling*2*d*(*MaxPooling*2*D*)	(None, 136, 181, 64)	0
*conv*2*d*_2(*Conv*2*D*)	(None, 136, 181, 128)	73856
*max*_*pooling*2*d*_1(*MaxPooling*2*D*)	(None, 68, 90, 128)	0
*conv*2*d*_3(*Conv*2*D*)	(None, 68, 90, 256)	295168
*max*_*pooling*2*d*_2(*MaxPooling*2*D*)	(None, 34, 45, 256)	0
*conv*2*d*_4(*Conv*2*D*)	(None, 34, 45, 512)	1180160
*max*_*pooling*2*d*_3(*MaxPooling*2*D*)	(None, 17, 22, 512)	0
*conv*2*d*_5(*Conv*2*D*)	(None, 17, 22, 512)	2359808
*max*_*pooling*2*d*_4(*MaxPooling*2*D*)	(None, 8, 11, 512)	0
*flatten*(*Flatten*)	(None, 45056)	0
*dense*(*Dense*)	(None, 1024)	46138368
*dense*_1(*Dense*)	(None, 1024)	1049600
*dense*_2(*Dense*)	(None, 25)	25625
**Total parameters**: 51,161,305		
**Trainable parameters**: 51,161,305		
**Non-trainable parameters**: 0		

#### Convolutional layer

The above stated proposed model used six convolutional layers. There are two types of characteristics in each layer, i.e., input and numeral filters. The filter numbers are then convolved on each layer which extracts the useful features and passes it to the next connected layer. For an RGB image, each filter is applied to all three colour channels, and thus, a corresponding matrix is obtained accordingly. We used a filter size of 3 x 3 for all convolutional layers. Pooling Layer: Most commonly, the pooling layer follows each convolutional layer. There are five max-pooling layers in the proposed method. The pooling layers are often used to minimise computational cost as it reduces the size of each convolutional layer output. The max-pooling has an activated filter which slides on the input and based on the size of the filter, and the max value is selected as an output. We used a filter of 2 x 2 for all max-pooling layer.

#### Dense layer

It is also known as an artificial neural network (ANN) classifier. Our model has three dense or fully connected layers. In fully-connected layers, each node is connected with only one node of another layer. The first two fully-connected layers have ReLu activation during the last layer, which is also known as the output layer, has a softmax activation. The softmax activation works by finding the node with the highest probability value of prediction being made. Hence the node with the higher value is forwarded as an output.

#### Dropout

The overfitting issue is prevented by the addition of a dropout of 0.5. It is added to the dense layers of the model.

#### Parameters

The total model parameters of our model are 51,161,305.

The model takes the image data as an input, then processes that input data by extracting features from the image and then classifies it either healthy or a diseased leaf, if it is an infected leaf then it further predicts the disease class name, the most resemble one. The expected class then results as an output.

We made changes to the hyper-parameters shown in the Table 3 to optimise our model. We selected the optimizer of Stochastic Gradient Descent (SGD), proving to be an optimal trade-off between accuracy and effectiveness [46]. The SGD is clear and reliable. The hyper-parameters model to be tuned, in particular the initial learning rate, which is used in optimization as it explains how rapidly the weights are altered to achieve a minimum of the local or global loss function. The momentum (= 0.9) tends to accelerate SGD in the correct direction and dampens the oscillations [47]. In addition, regularisation is a very effective method to avoid over-fitting. The most common way of regularisation is L2 Regularization, where the combination with SGD results in weight decay, in which the weights for each update are scaled by a factor slightly smaller than one [48]. A total of 50 epochs are performed in each experiment, where each epoch is the number of training iterations. Finally, DCDM is trained at a batch size of 32 and stopped training on epoch-50.

**Table 3:** Hyper-parameters of the experiments.

Hyper-Parameters	Value
Optimizer	SGD
Momentum	0.9
Epochs	50
Batch Size	32
Dropout rate	0.5
No. of Layer	9
Learning Rate	1.0*x*10^−3^
Loss Function	Cross Entropy

### 2.5 Transfer learning in AWS cloud

Transfer learning (TL) is a concept in the ML which simply means that a method learns basic knowledge in solving a particular problem and later reusing that knowledge for other more or less similar problem solution [49]. This technique encourages us to use for solving any relevant problem for which there is not sufficient data available. Thus it relaxed the assumption of training and testing data, should be both distributed identically and independently [50]. It takes a long time and large-sized dataset for training CNN from scratch. Hence in certain situations where the dataset is limited, then TL is a helpful method. We used TL from scratch for DCDM training. Amazon’s Cloud platform and AWS DeepLens were selected to address the scalability constraints. The Amazon cloud infrastructure offers data collection, data transfer and computing resources for the application development and deployment. AWS provides many services and several different applications. They also have a platform for building, preparation and rollout, as well as validating models of machine learning. On AWS Services or some other compatible systems, for example, AWS DeepLens, the trained model can be deployed.

### 2.6 Lambda function on DeepLens

**Fig 6:**
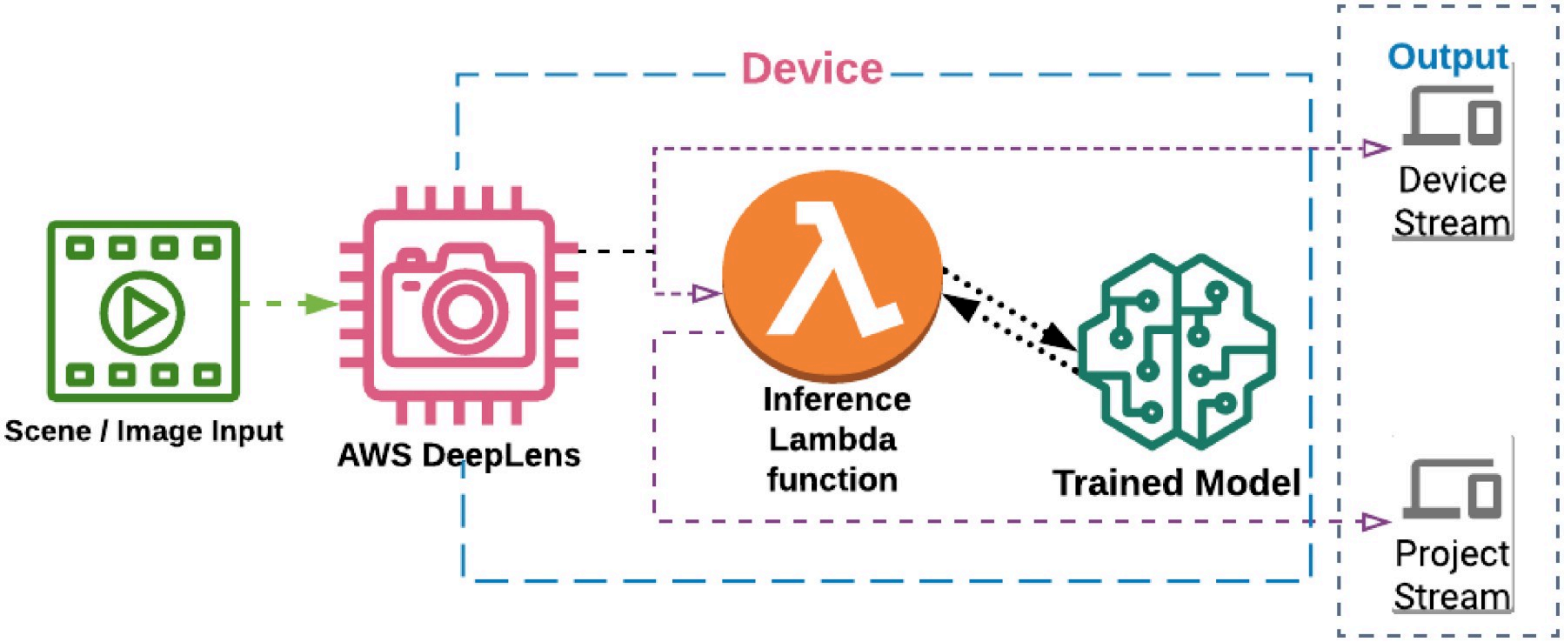
Basic workflow of a deployed AWS DeepLens project [55].

AWS DeepLens is a deep-learning-based high definition (H.D), 4-mega-pixel video camera that is designed specifically for machine learning models developments and implementation. It has a built-in 8GB memory and 16GB storage capacity with 32 GB SD card (extendable). It has more than 100 GFLOPS computing power so it can process machine learning projects independently as well as those integrated with AWS Cloud [51]. It has a straightforward usage process as the user can take picture/ image through DeepLens camera, then store it and process it to use in machine learning projects [52]. There are a large number of pre-trained models, built-in to it, but a customised model can also be used with DeepLens camera. For instance, any custom based model can be trained or imported into in SageMaker and then can be implemented in AWS DeepLens through various deep learning frameworks such as Tensorflow or Caffe [51, 52]. A lambda function is used to establish a successful connection to access the DeepLens on a local computer. The lambda functions are the pre-defined functions executed by DeepLens once the project has been deployed [53]. Lambda function streamlines the development process by managing the servers necessary to execute code. They serve as the connection between the AWS DeepLens and Amazon Sagemaker for the camera to generate a real-time inference [54]. It controls various resources such as computing capability and power, networking. It has a user-specified function embedded in code, and Lambda function invoke that user code when it is executed. The code returns a message containing data from the event received as input [54]. The visual illustration of the AWS DeepLens work-flow is shown in Fig 6.

After completing the training stage in SageMaker, we implemented the subsequent trained Model in AWS DeepLens camera for inferences of Leaves health assessment.

### 2.7 Evaluation and performance measurement

Several methods are used to measure the efficiency of neural networks, including precision, recall, accuracy, and f1-score. The precision tells us about the correct predictions made out of false-positive while recall tells us about the accurate predictions made out of false negatives. The accuracy is the number of correct predictions out of both false positives and false negatives. All the performance metrics for our trained model have been determined using the formulas in Eqs (1), (2), (3) and (4) are listed. We calculated the values from the confusion matrix shown in Fig 10.

$$\begin{array}{c} Precision=\frac{TP}{TP+FP} \end{array}$$ (1)

$$\begin{array}{c} Recall=\frac{TP}{TP+FN} \end{array}$$ (2)

$$\begin{array}{c} Accuracy=\frac{TP+TN}{TP+TN+FN+FP} \end{array}$$ (3)

$$\begin{array}{c} F1-Score=2*\;\frac{precision\;*\;recall}{precision+recall} \end{array}$$ (4)

Where TP is true positives, TN is true negatives, FP is false positives and FN is false negatives. Here the TP and TN are the correct predictions while the FP and FN are the wrong predictions made by our model.

### 2.8 Features maps extraction and filters visualization in CNN layers

#### 2.8.1 Extraction of feature maps

**Fig 7:**
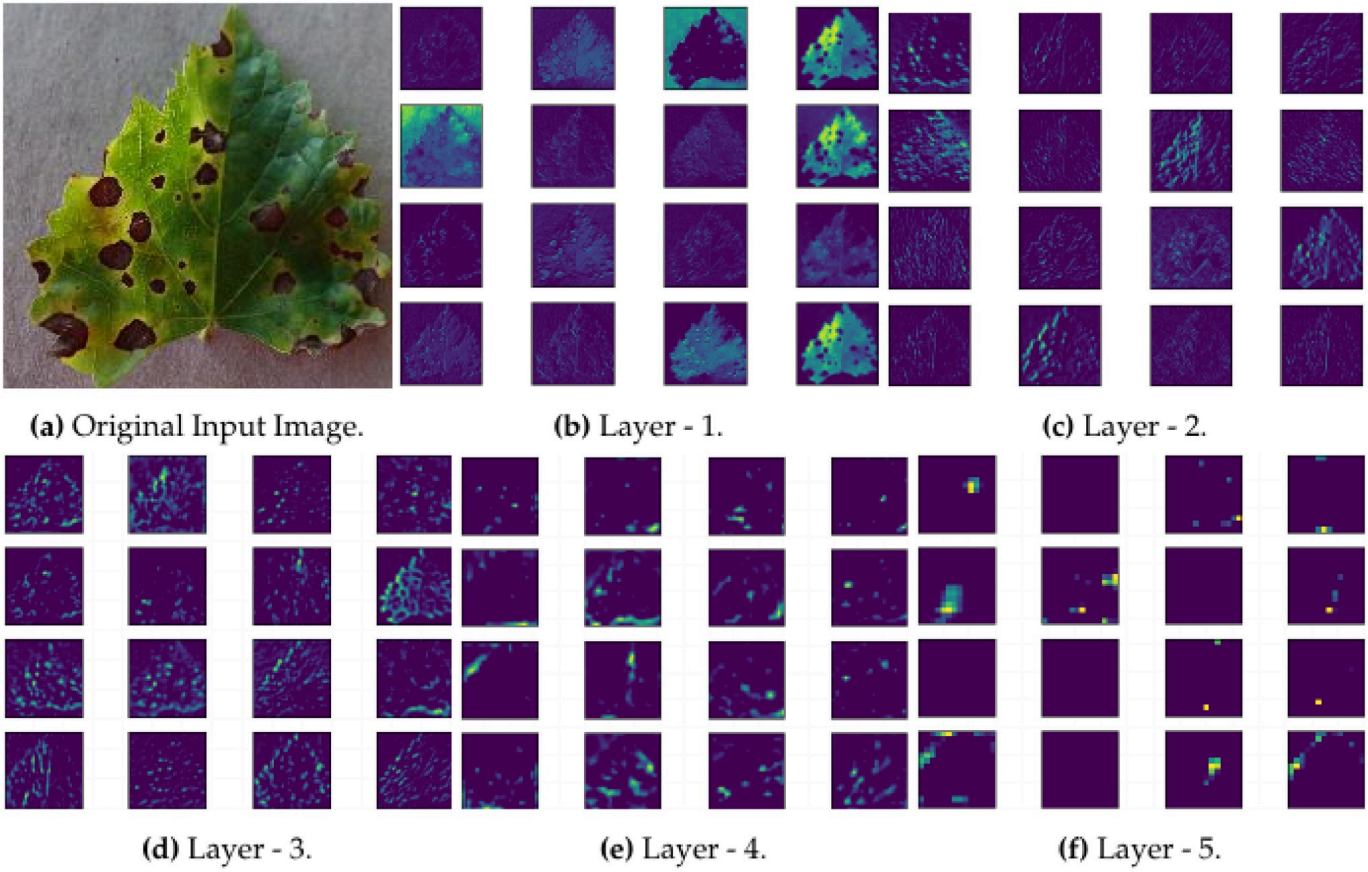
Visualization of feature map from DCDM convolutional layer for a sample leaf.

Feature maps [56] are used to present the local information passing through the CNN Layers. In an ideal feature mapping of CNN, they are sparse and help in the understanding of the classical model. In convolutional layer, to extract feature maps from the source image, several mathematical computations are carried out [57]. In Fig 7, a visual representation for the extraction of feature maps presented for various layers of our model. It also provides information about each layer, i.e. what and how a particular layer of CNN gains information from other layers, such piece of information can help the developer to make proper adjustments in the developing model for best results. From our visualisation images, we found that our model is gaining information in the hierarchical order. It means that the high-level layers present more specific features and vice versa.

Similarly, if the dimensions are higher than feature maps, images would also be more accurately classified. For instance, in an image, the edge corners and some abstract colour features are presented by a deep layer Fig 7, while other corners and edges represented in shallows layers. Moreover, the middle layers are usually responsible for capturing the same textures because these layers are having complex invariance and more layers in number, after extracting higher-level abstract features, the striking posture of the entire image shown by the high-level feature map.

The feature maps extracted in the first layer represents the overall physical appearance of the leaf image. In the middle layers, the patterns of disease are extracted as can be seen in Fig 6. The last layers in Fig 6 often extract the delicate features as they are then used to finalise the predicted class.

### 2.9 Filter visualization in model layers

**Fig 8:**
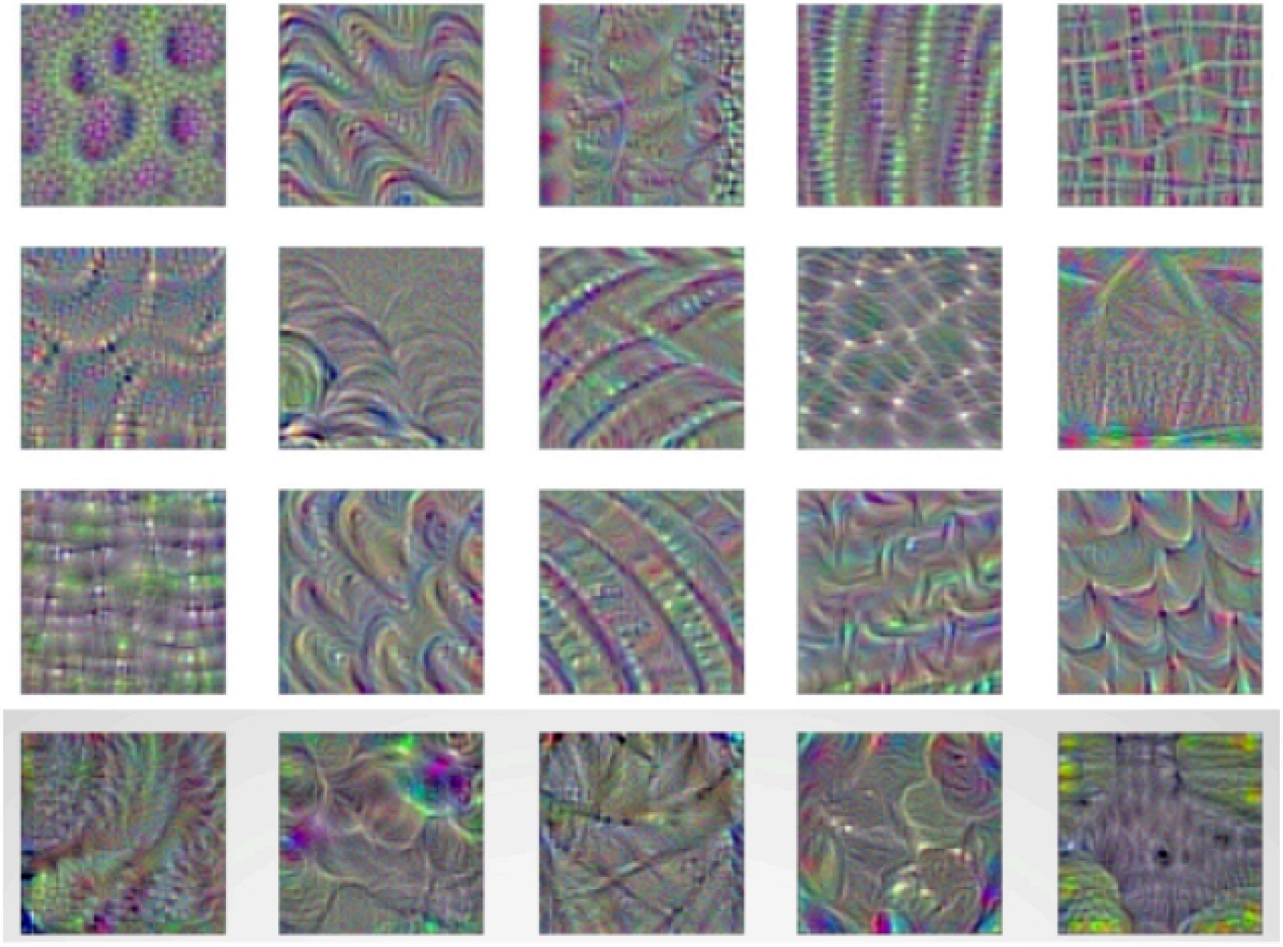
Visualisation of filter activation in DCDM convolution layers.

Generally, filters are used for the detection of unique patterns in an input image. It is done by detecting the change in the intensity values of the image. Thus, each filter has its particular importance for feature extraction [58]. As an example, a high pass filter detects the existence of edges in an image. In our DCDM model, various filters are used to extract features like edges, shape, the colour of the leaf, and many more useful features. In Fig 8, a visual representation for a few filters presented where each filter has its application for extracting leaf features. After detecting the specific feature of the image by a filter, it is then passed to the next layer where other filters extract the additional feature. This process continues until the last layer, and thus integrating all together helps to define the predicted class for an input image.

## 3 Experimental results

The entire dataset was distributed into different training sets (80%, 70% and 60%) and testing data (20%, 30% and 40%) for performance evaluation, as shown in Table 4. The model was using 10% of the each training set split for validation purpose during its training phase.

**Table 4:** Dataset split for training and testing.

Train—Test Data Split (%)	Training Images	Testing Images
80—20	40000	10000
70—30	35000	15000
60—40	30000	20000

The performance indicator, accuracy for each data split is shown in the Table 5. After every ten epochs of preparation, the values are presented. However, in comparison with another train-test dataset splits for DCDM model performance evaluation, the data split of 80%—20% performed very well at the epoch scale of 50 with the maximum accuracy of 98.78% as shown in Table 5.

**Table 5:** Dataset split for training/testing and accuracy obtained per epoch.

Dataset (Train/Test) Split in %	Accuracy [%]				
	10 Epochs	20 Epochs	30 Epochs	40 Epochs	50 Epochs
80–20	92.31	95.84	96.86	97.39	**98.78**
70–30	91.23	94.89	96.15	96.77	97.46
60–40	90.70	94.92	95.04	95.98	96.21

**Fig 9:**
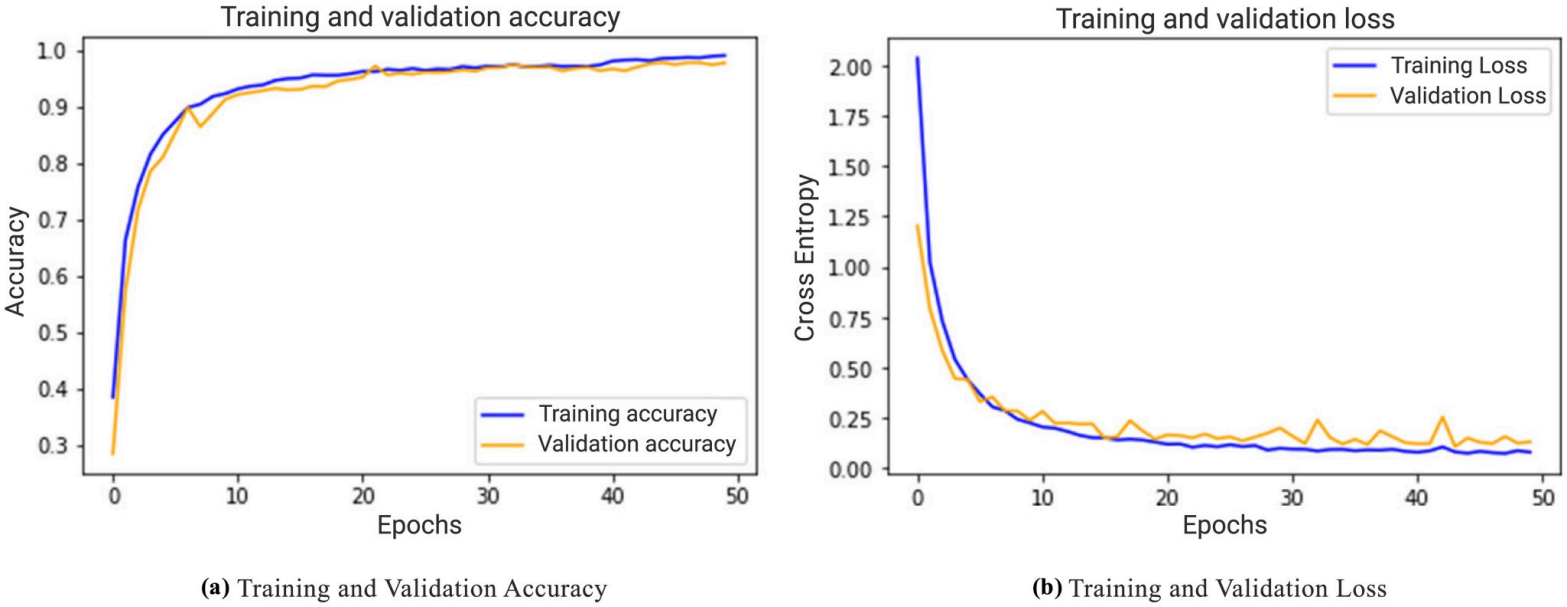
Trend graph for accuracy and loss in training and validation.

In Fig 9(a) and 9(b), the accuracy and loss for both training and testing/validating are presented for each epoch. These graphs were generated for the data split of 80%–20%. The accuracy graph visually shows that accuracy increases gradually for both training and testing, and then tends to converge on a specific point. It also shows that after 40 epochs, the change in accuracy reduces as the validation accuracy appears to be equivalent to training accuracy. Similarly, the right graph shows how the loss starts decreasing gradually as the model learns on a given dataset. The loss of validation data becomes stable after 43 epochs and thus tends towards a specific value.

**Fig 10:**
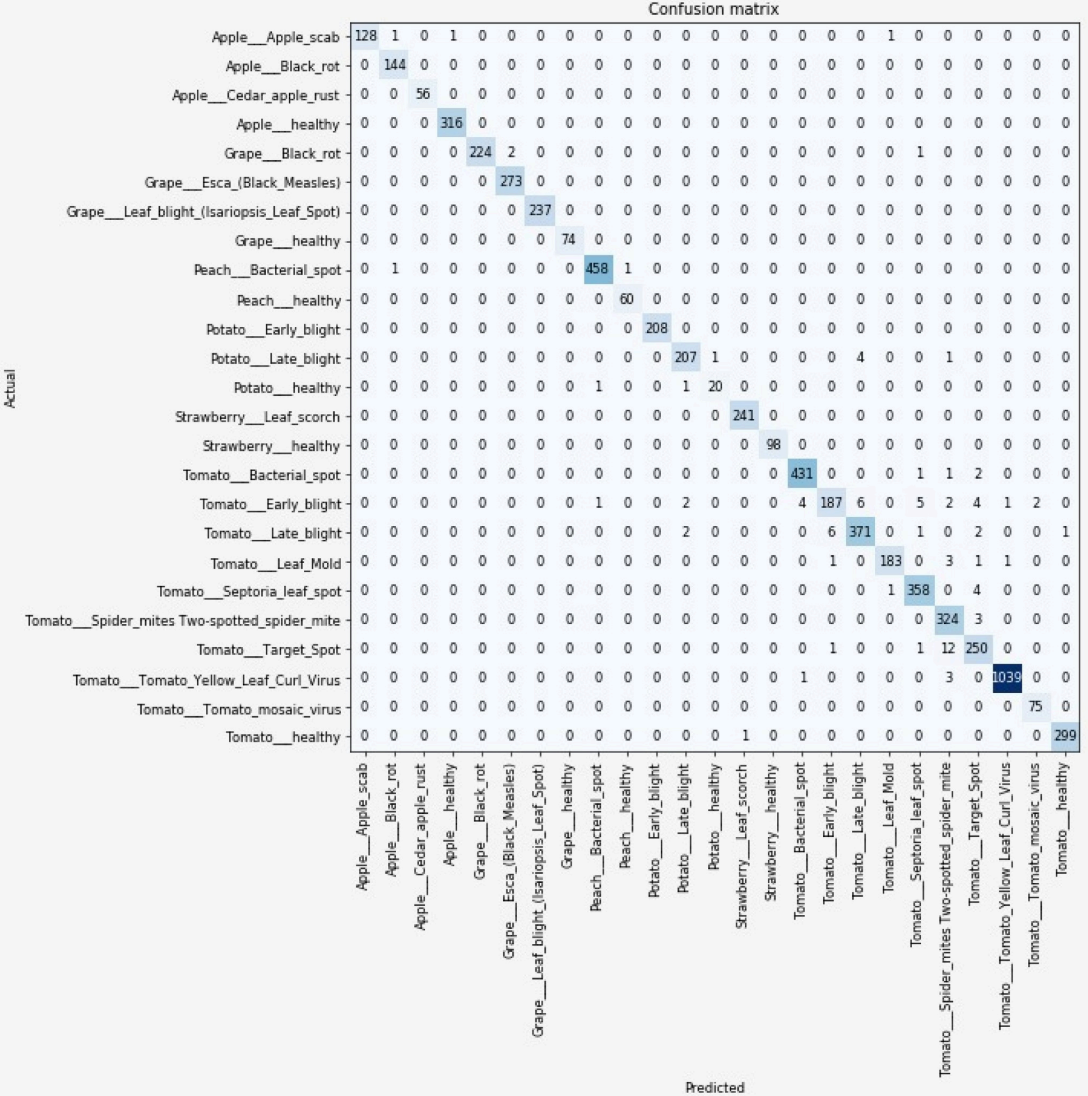
Confusion matrix for 80-20% dataset split set.

The validation process gives a confusion matrix shown in Fig 10. After computing values from the confusion matrix, the results are shown for the 80%-20% split ratio in Table 6.

**Table 6:** DCDM performance report.

Evaluation Metrics	Value in %
Precision	98.38%
Recall	97.98%
Accuracy	98.78%
F1-Score	98.17%

The confusion matrix shows the predictions made by 80-20 dataset split are presented in the Fig 10. The matrix displays the number of predictions that are true and false. It also offers the information for which class is more reliably predicted and vice versa. The groups of Apple Cedar Rust, Grape Leaf Blight, Grape Healthy, Potato Early Blight, and Strawberry Healthy have been correctly predicted, so these groups have not been wrongly predicted. While the Tomato Early Blight, Tomato Late Blight, Tomato Spider Mites, and Tomato Goal Spot classes have the most inaccurate predictions from other classes. Likewise, the Potato Late Blight and Tomato Septoria Leaf Spot groups have an average number of false predictions. The remaining groups are expected with a minimum number of incorrect predictions, such as Apple Scab, Apple Black Rot, Apple Safe, etc.

**Fig 11:**
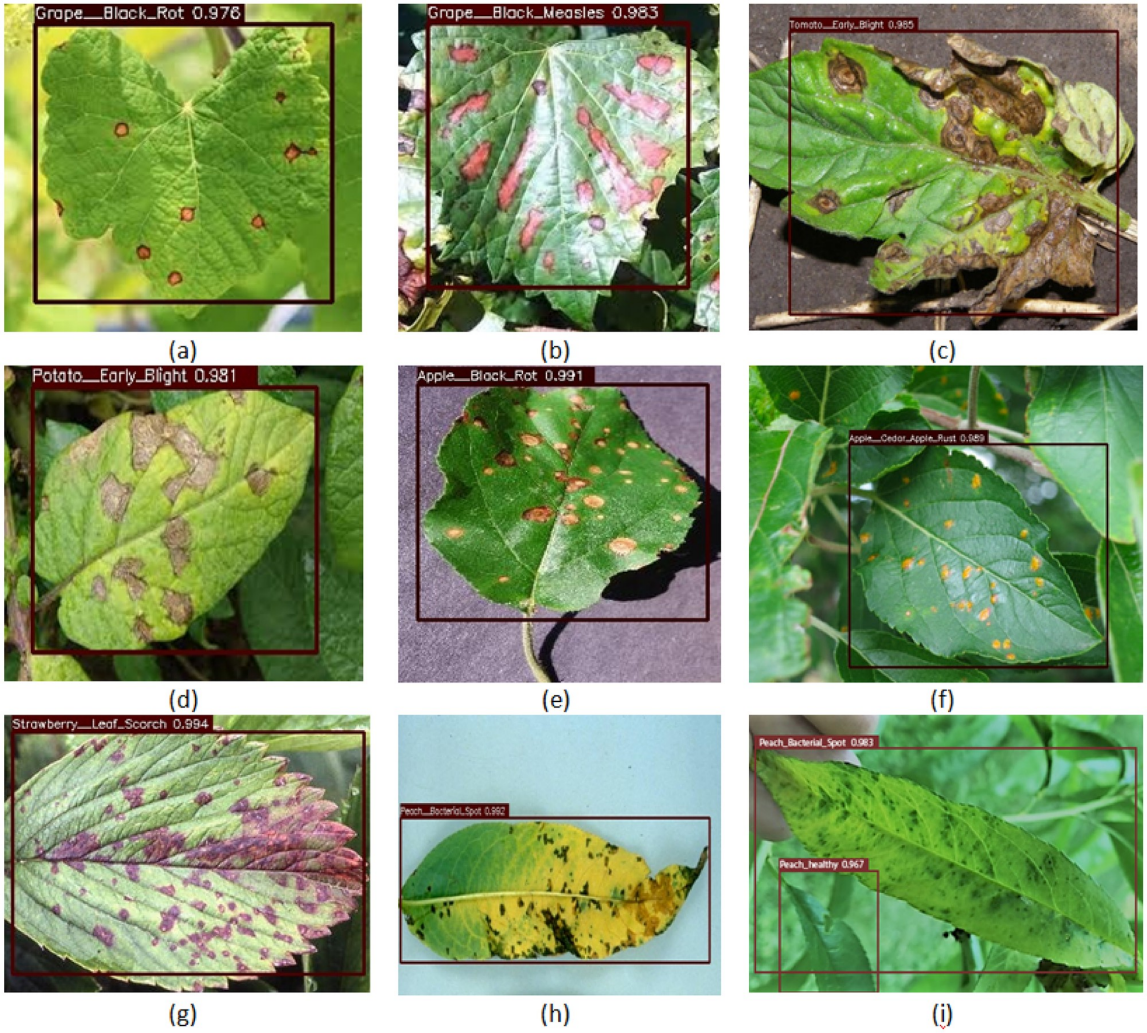
Sample results from real field and controlled environment images.

Some of the sample output images with an AWS DeepLens are shown in Fig 11.

### 3.1 Comparative analysis

A comparative overview of different CNN architectures with the DCDM, is given in this section. Training the model on different architectures is a crucial approach used to define the best architecture for targeted application. The architectures we used for the identification and classification problem are the highest performing architectures. We compared the performance of DCDM, ResNet-50 [31], DensNet [33], VGG-16 [15], VGG-19 [15], AlexNet [32], SqueezeNet [34] and DarkNet [35] architecture for each training and testing dataset split using same hyper-parameters. An evaluation metric of accuracy was used for comparison, based on Eq 3.

**Fig 12:**
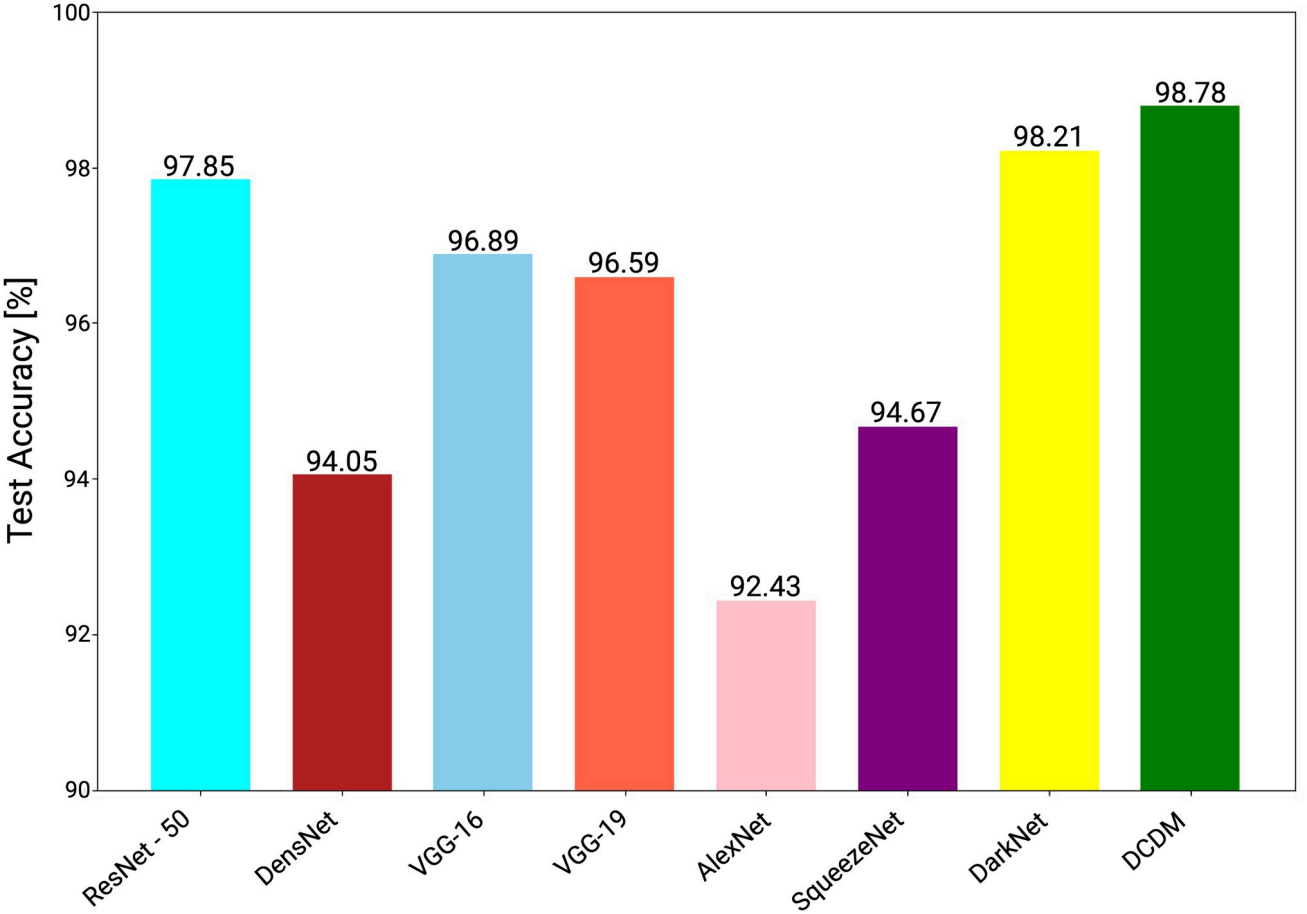
Average accuracy obtained by each CNN model.

For each CNN architecture, we obtained an output accuracy of more than 90%. AlexNet architecture works with the lowest precision of 92.43%. This architecture is considered to be the smallest and most simple architecture of all. However, it still provides us with a accuracy of over 90%. With some slight changes and a different number of layers, the VGG-16 and VGG-19 architectures are the same. For the classification challenges, they have an important record of doing very well. They provide us with an accuracy of 94.05% and 96.89% respectively for our research dataset. Similarly, SqueezeNet and DenseNet architecture also performed with an accuracy of 94.67% and 96.59%. The ResNet-50 architecture is well-known for good performance on large datasets. It has a bulk of 50 layers with different inter-connections. Thus, performing with an accuracy of 97.85% and being able to score the position of the third-best architecture in our list. The architecture of DarkNet provides an accuracy close to DCDM model. It results from the accuracy of 98.21%, scoring the position of second-best architecture while DCDM architecture performed outstanding and stood with the position of best architecture with an accuracy of 98.78%. The results for each architecture based on accuracy is visually represented in Fig 12.

The comparison of each architecture concerning time consumed has also been made which implies the time required for training. The time consumed by our architecture requires less computation time, thus having the lowest average time during training per epoch. It testifies that our architecture is the most efficient both performance as well as computation wise. The results for each architecture on the basis of computation time taken per epoch is shown in Table 7.

**Table 7:** Average time consumed by CNN’s per epoch.

Trained CNN Models	Average Time Per Epoch (in Minutes)
ResNet-50	2:03
DensNet	2:38
VGG-16	1:53
VGG-19	1:59
AlexNet	1:44
SqueezeNet	2:32
DarkNet	2:13
DCDM	**1:26**

The DCDM model realises higher convergence speed and greater accuracy during the training phase relative to the regular CNN architectures (ResNet-50, VGG-16, VGG-19, DensNet, AlexNet, SqueezeNet, DarkNet, etc.). The findings of this study show that end-to-end classification of plant leaf diseases is realised by the proposed algorithm and offers a solution and a reference for the implementation of deep learning approaches in plant disease classification.

## 4 Discussion

The conventional approach to image classification methods focused on hand-engineered features such as SIFT [59], HoG [60] and SURF [61] etc., has previously been used to remove features from pictures. Thus, relying heavily on the pre-defined features underlying them, added to the success of all these methods. Function descriptors themselves are a complicated and repetitive process that may be revisited if there is a substantial change in the topic at hand or the parameters of the corresponding dataset. In all conventional attempts to diagnose plant diseases utilizing image recognition, this difficulty has arisen because they rely solely on hand-engineered features, techniques of image enhancement, and a variety of several other challenging and exhaustive methodologies [36].

DL has significantly advanced in many research areas. Deep Neural Network for Convolution (CNN) Architectures [62] have become famous recently as they eliminate the dependency on explicit hand-crafted features instead, learn strong feature representations directly, from raw data. The integration of these deep neural networks features at different specificity levels (ranging from low-level functions such as edges to abstract high-level features such as objects) [63] and comprehensive classifiers, fashion from end-to-end. Indeed, the architecture of Deep CNN has state-of-the-art performance obtained on image classification tasks [64, 65].

We find that most of the images in the PlantVillage dataset are either white or grey background, but the real-world situation is different and can include other background colours. Thus, the only uniform background colour trained in the model will result in low accuracy of false prediction. To achieve high accuracy and a stable model, we used a mix of PlantVillage dataset and images gathered from Tarnab Farm, Pakistan the real-cultivation and research environment. We applied various data augmentation techniques to the training data to maximize the number of those leaf disease classes where they were less in number. Thus the processed dataset comprised of around fifty thousand images of twenty-five different infected and healthy plant leave classes from six plants i.e. apple, grapes, peach, strawberry, potato and tomato.

We proposed a DeepLens Classification and Detection Model (DCDM) to recognise and diagnose multiple fruit trees and vegetable plant leaf diseases. We used a cloud-based environment for DCDM training and testing to address the concerns of scalability and applicability. It was deployed on AWS DeepLens after completion of DCDM training. For ML projects, AWS Deeplens is a DL-based camera with a 4 mega-pixel high definition (HD) sensor.

We compared DCDM with seven different CNN architectures utilising performance accuracy and computation time. ResNet-50 [31], AlexNet [32], VGG-16 [15], VGG-19 [15], DenseNet [33], SqueezeNet [34] and DarkNet [35] are included in these CNN architectures. All of these models have been trained and tested under the same environment, i.e. same dataset set was used for training and testing phases using the same hyper-parameters for all. All other architectures exceeded our DCDM model in terms of computing time, as shown in Table 7. On real field and test images, DCDM obtained an overall accuracy rate of 98.78%, which is higher than others as shown in Fig 12. Our study findings are the first step towards a system for plant disease diagnosis based on an AWS DeepLens camera.

At the current point, however, there are a range of weaknesses that need to be dealt with in future work. Firstly, in addition to AWS Deeplens, it can be easily implemented in the future on multiple mobile platforms such as iOS, Android or Windows-based mobile applications due to the fast classification process of our model. Secondly, More plant species will be introduced to make this model more scalable in the future. As there are few plant species at present, they are included and evaluated. Lastly, in future work, modern techniques such as Multi-spectral and Hyper-spectral images should also be tested for the detection and classification of plant diseases.

## 5 Conclusion

With this proposed deep model applied on AWS DeepLens, 25 separate disease classes in Apple, Grape, Peach, Potato, Strawberry and Tomatoes can be predicted in real-time. In real-time predictions and classifications for field experiments, our model gained 98.78% accuracy. This practical method would facilitate the practitioners and society relevant to agriculture by contributing to the enhancement of the agri-economy, as the severe issue of plant (leaves) diseases, can be instantly recognised and classified. In addition, this approach is scalable, and it can also be used as an online repository for plant leaves disease identification and classification. More classes of other vegetables and fruit leaves can also be added in future. To improve its usability and applicability, we will incorporate our model into various mobile platforms such as iOS, Windows and Android-based applications in our future work. Thus, due to regular smartphone use, the functionality would become more flexible and easy to use. Moreover, new techniques such as multi-spectral and hyper-spectral images should also be evaluated in future work for the identification and classification of plant diseases.
